# Melatonin Protects Against Neuronal Apoptosis via Suppression of the ATF6/CHOP Pathway in a Rat Model of Intracerebral Hemorrhage

**DOI:** 10.3389/fnins.2018.00638

**Published:** 2018-09-19

**Authors:** Weilin Xu, Xiaoyang Lu, Jingwei Zheng, Tao Li, Liansheng Gao, Cameron Lenahan, Anwen Shao, Jianmin Zhang, Jun Yu

**Affiliations:** ^1^Department of Neurosurgery, Second Affiliated Hospital, School of Medicine, Zhejiang University, Hangzhou, China; ^2^Burrell College of Osteopathic Medicine, New Mexico State University, Las Cruces, NM, United States; ^3^Brain Research Institute, Zhejiang University, Hangzhou, China; ^4^Collaborative Innovation Center for Brain Science, Zhejiang University, Hangzhou, China

**Keywords:** intracerebral hemorrhage, mesencephalic astrocyte-derived neurotrophic factor (ATF6), CCAAT/enhancer-binding protein homologous protein (CHOP), apoptosis, secondary brain injury

## Abstract

Neuronal apoptosis is an important factor accounting for the poor outcomes of intracerebral hemorrhage (ICH). This study first showed that inhibition of activating transcription factor 6 (ATF6) could alleviate secondary brain injury through anti-apoptosis after ICH in rats. Melatonin, ATF6 and CCAAT/enhancer-binding protein homologous protein (CHOP) siRNAs were applied in this study. Brain edema, neurological functions, blood-brain barrier (BBB) integrity were evaluated at 24 h after ICH. Western blot analysis was used to evaluate the protein level of target proteins (ATF6, CHOP, Bip, Bcl-2, Bax, and cleaved caspase-3). Reverse transcription-polymerase chain reaction (RT-PCR) was used to assess the mRNA level of ATF6, CHOP and cleaved caspase-3. Terminal deoxynucleotidyl transferase-mediated dUTP nick end labeling (TUNEL) and caspase-3 immunofluorescence staining were applied to evaluate the neuronal cell death. The results suggested that the levels of ATF6 and its downstream protein, CHOP, were upregulated and reached the peak at 24 h after ICH. ATF6 was highly expressed in neurons. The administration of melatonin significantly decreased the mRNA and protein levels of ATF6, and its downstream targets, CHOP and cleaved caspase-3, but increased the Bcl-2/Bax ratio, which ameliorated the neurological functions. The CHOP siRNA significantly reversed the pro-apoptotic effect induced by the increased ATF6 level after ICH. Melatonin could protect against neuronal apoptosis via suppression of ATF6/CHOP arm of ER-stress-response pathway.

## Introduction

Intracerebral hemorrhage is one of the most common types of stroke (Feigin et al., 2009; Steiner et al., 2014), which is featured by a high rate of death and disability (van Asch et al., 2010; Krishnamurthi et al., 2014). The underlying mechanisms accounting for the poor prognosis after ICH include neural apoptosis, neuroinflammation, oxidative stress, et al (Shen et al., 2017; Zeng et al., 2017). Among them, apoptosis plays a pivotal role in the secondary brain injuries. Surgical procedures are restricted and can only relieve a small number of patients (Pías-Peleteiro et al., 2017). Many studies focus on the pharmacological treatment of ICH, but no target has been launched with specific efficacy.

The ER is an organelle which mainly provides a topologically distinct membranous network for protein modification, proper protein folding and assembly (Senkal et al., 2010). Insults that perturb ER function result in ER stress (Roussel et al., 2013). The pathophysiological process of inflammation, oxidative stress, mitochondrial calcium overloading (Zhou et al., 2015), and toxic glutamate release induced by ICH could greatly activate the ER stress response. Finally, the overloaded proteins could further trigger UPR (Schroder and Kaufman, 2005). The UPR is mainly conducted via three types of ER stress sensor proteins, IRE1, RNA-activated PERK, and ATF6. Among the three, ATF6 was reported to display the key roles in the UPR (DuRose et al., 2006; Wu et al., 2007; Doroudgar and Glembotski, 2012).

Long-lasting and serious ER stress was reported to cause cell death (Tabas and Ron, 2011; Fouillet et al., 2012). Once the ER stress is over activated, protein synthesis outpaces protein-folding capacity, then ATF6 moves to the Golgi, and is cleaved by the Golgi-localized proteases, SP1 and SP2 (Ye et al., 2000). The resulting 50 kD cytosolic fragment translocates to the nucleus, binds to elements in ATF6-responsive genes, and up-regulates its downstream target, CHOP. CHOP is a transcriptional factor and is involved in the commitment phase of ER stress-mediated apoptosis (Li et al., 2014). Suppression of ATF6 was also reported to provide neuroprotection in a cerebral ischemic model (Menzie-Suderam et al., 2018), but the role of ATF6 has not yet been explored in ICH.

Furthermore, as ATF6 is one of the main proteins in the UPR, we hypothesized that melatonin conducted its neuroprotective effects via suppressing the activation of ATF6/CHOP pathway. The neuroprotective effects of melatonin have been widely verified. In recent years, many studies put the focus on the effects of melatonin on ER stress (Fernández et al., 2015; Lin et al., 2018). Their results suggested that melatonin could exert its neuroprotective effects via suppression of ER stress.

In this study, we verified the following hypotheses: (1) ICH resulted in an increase of ATF6 expression; (2) the suppression of ATF6 by melatonin reduced brain edema, alleviated BBB disruption, and improved neurological functions; (3) selective inhibition of ATF6 expression with siRNA protected neuronal cells from apoptosis via suppressing the expression of CHOP and other pro-apoptotic factors, Bax and cleaved caspase-3.

## Materials and Methods

### Animals

We did all the operations according to the Institutional Animal Care and Use Committee of Zhejiang University. The procedures were conducted according to the National Institutes of Health’s Guide for the Care and the Use of Laboratory Animals and the ARRIVE (Animal Research: Reporting *in vivo* Experiments) guidelines. We only chose Sprague–Dawley (SD, Male) rats (291 rats, 280–330 g) (SLAC Laboratory Animal Co., Ltd. Shanghai, China) in this study (**Supplementary Table I**). We kept the rats in a 12 h day/night cycle (22 ± 1°C; 60 ± 5% humidity). The rats were free to water and food.

### ICH Rat Model

The ICH model was performed as previously described (Zhou et al., 2014). We used pentobarbital (40 mg/kg, intraperitoneal injection) to anesthetize the rat and then immobilized it in a stereotaxic frame (Stoelting Co., United States). Briefly, the right femoral artery was exposed and we obtained blood (100 μl) for the following injection. Then, we used cranial drill to make a burr hole 3.5 mm lateral right of the bregma. Afterward, the blood was manually injected in the right striatum (5.5 mm depth). We withdraw the needle 10 min after injection. Finally, we used medical bone wax to seal the burr hole and closed the incision with sutures. For the rats in sham group, we did all the procedures except for the injection.

### Experimental Design (**Figure 1**)

**FIGURE 1:**
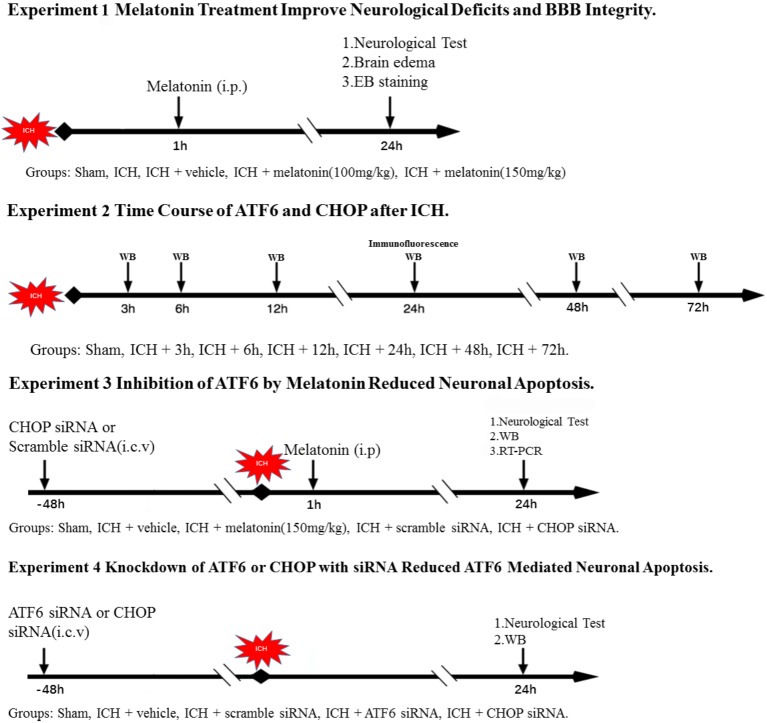
Experimental design and animal groups.

In the first step, we explored the effects of melatonin. Sixty rats were distributed into five groups: sham (*n* = 12), ICH (*n* = 12), ICH + vehicle (*n* = 12), ICH + melatonin (100 mg/kg, *n* = 12), ICH + melatonin (150 mg/kg, *n* = 12). We assessed neurobehavior conditions, brain edema (*n* = 6), and EB extravasation (*n* = 6) at 24 h after ICH in each group.

In the second step, the time course of ATF6 and CHOP was evaluated in sham group and ICH groups with different time points. Forty-six rats were randomized distributed into seven groups: sham (*n* = 8), 3 h (*n* = 6), 6 h (*n* = 6), 12 h (*n* = 6), 24 h (*n* = 8), 48 h (*n* = 6), and 72 h (*n* = 6). Western blot analysis was performed in each group (n = 6). Two rats in the sham and ICH(24 h) were used for colocalized immunofluorescence staining of ATF6 with neuronal nuclei (NeuN).

In the third step, in order to further explore the underlying mechanisms of neuroprotective effects of melatonin, ninety-six rats were distributed into five groups: sham (*n* = 20), ICH + vehicle (*n* = 20), ICH + melatonin (best dosage, *n* = 20), ICH + scramble siRNA (500 pmol in 5 μl in sterile saline, *n* = 18), or ICH + CHOP siRNA (500 pmol in 5 μl in sterile saline, *n* = 18). The siRNA was injected intracerebroventricularly at 48 h before induction of ICH. Six rats per group were used for Western blot analysis and RT-PCR, respectively. Furthermore, five rats in each group were used for TUNEL and caspase-3 staining.

In the fourth step, we adopted ATF6 siRNA to further verify the mechanisms mentioned above, as melatonin is not a selective inhibitor of ATF6. Sixty rats were distributed into five groups: sham (*n* = 12), ICH + vehicle (*n* = 12), ICH + scramble siRNA (500 pmol in 5 μl in sterile saline, *n* = 12), ICH + ATF6 siRNA (500 pmol in 5 μl in sterile saline, *n* = 12), and ICH + CHOP siRNA (500 pmol in 5 μl in sterile saline, *n* = 12). The siRNA was injected intracerebroventricularly at 48 h before induction of ICH. Six rats per group were used for Western blot analysis and RT-PCR, respectively.

### Behavioral Assessment

Neurobehavior conditions were assessed at 24 h after ICH based on a system named the NSS (Cui et al., 2017). Detailed information about the marking system is displayed in **Supplementary Table II**.

### Brain Water Content

We used wet–dry method to evaluated the brain water content at 24 h after ICH. Briefly, after euthanasia, we collected the brain hemisphere and weighed it (wet weight). Then, right hemisphere of the brain was put in an oven for 72 h (105°C, dry weight). Finally, we calculate the brain water content as follows: [(wet weight - dry weight)/(wet weight)] × 100% (Chen et al., 2015).

### Evans Blue (EB) Staining

Evans blue staining was applied to evaluate the blood–brain barrier integrity. 2% EB solution (8 mL/kg, Sigma–Aldrich) was intra-peritoneally injected after anesthetization. After 24 h, the rats received trans-cardiac perfusion with 0.1M PBS. Next, the brain was removed and homogenized in 50% trichloroacetic acid. The sample was incubated in a water bath (50°C) for 48 h and centrifuged at 15,000 × *g* for 30 min. Afterward, the supernatant was detected through spectro-fluorophotometry at 620 nm (Zhao et al., 2016).

### Immunofluorescence and Calculation of Apoptotic Cells

After anesthetization, transcardial perfusion with 0.1M PBS was performed, followed by another perfusion with 4% paraformaldehyde (pH = 7.4). Then, the cerebral hemispheres were removed and put into 4% PFA for post-fixation (4°C, 24 h). Afterward, the brains were transferred to sucrose solution (30%, 2 days). Next, the brains were coronally sliced into 10 μm sections, which were then fixed on slides and used for immunofluorescence staining, and then blocked with 10% normal donkey serum for 2 h at room temperature and incubated at 4°C overnight with primary antibodies: ATF6 (1:200, abcam ab203119), caspase-3 (1:200, abcam ab49822), NeuN (1:500, abcam ab104224). Additionally, secondary antibodies were applied for 2 h at room temperature. Finally, the sections were assessed with a fluorescence microscope (Olympus, Tokyo, Japan) and pictures were further processed by Photoshop 13.0(Adobe Systems Inc., Seattle, WA, United States). Additionally, TUNEL (Roche Inc., Basel, Switzerland) staining was applied to assessed cellular apoptosis of brain tissues.

### Western Blot Analysis

**FIGURE 2:**
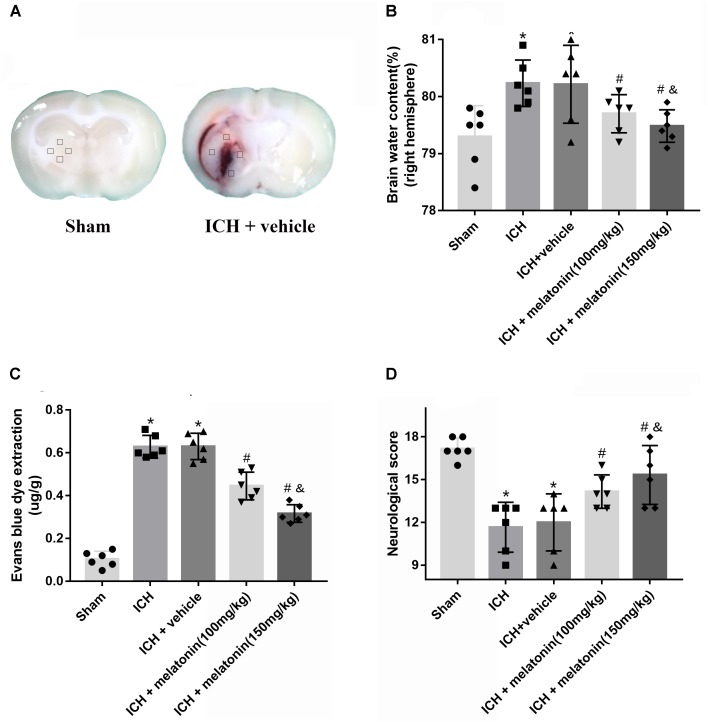
Effects of melatonin on neurological functions, brain edema and BBB leakage. **(A)** Representative pictures of brain slices in sham and ICH group (24 h); **(B)** the quantification of brain water content at 24 h after ICH; **(C)** the quantification of Evans blue dye extravasation at 24 h after ICH (*n* = 6 for each group); **(D)**. The quantification of neurological functions; The bars represent the mean ± SD. ^∗^*p* < 0.05 vs. sham, ^#^*p* < 0.05 vs. ICH at 24 h, ^&^*p* < 0.05 vs. ICH + melatonin (100 mg/kg).

First, the rats received trans-cardiac perfusion with 0.1M PBS after being anesthetized, then we collected the brain tissues around hematoma (**Figure 2A**), which were further processed as previously reported (Nakka et al., 2010), and 40 μg of protein from each sample was used for electrophoresis (100 V, 1 h) and then transferred to the polyvinylidene fluoride membranes at 250 V for 1 h. Afterward, the protein was incubated with primary antibodies overnight (4°C): ATF6 (1:500, abcam ab203119), CHOP (1:1000, abcam ab11419), Bip/GRP78 (1:2000, abcam ab21685), MMP-9 (1:800, SCBT, sc-12759), β-actin (1:5000, abcam ab8226), Bax (1:1000, abcam ab32503), Bcl-2 (1:500, abcam ab59348), and caspase-3 (1:500, abcam ab49822). After that, the proteins were incubated with secondary antibodies (1:10000, Zhongshan Gold Bridge) for 1 h. Finally, the ECL Plus chemiluminescence reagent kit (Amersham Bioscience, Arlington Heights, IL, United States) was used to detected the proteins. We displayed the results as the relative density which is the ratio of the grayscale value of the target proteins to that of β-actin.

### Small Interfering RNA and Intracerebroventricular Injection

The intracerebroventricular injection was conducted as previously reported (Zhou et al., 2018). After the rats were anesthetized, we used cranial drill to make a burr hole at 1 mm posterior to bregma and 1.5 mm right lateral to midline. Afterward, following the manufacturer’s instructions, a total volume of 5 μl (500 pmol) of rat ATF6 siRNA or CHOP siRNA (Thermo Fisher Scientific, United States) dissolved in nuclease-free water was injected into the right ventricle (3.5 mm depth below the skull) with a pump at the rate of 0.5 μl/min at 48 h before ICH. The same volume of scramble siRNA (Thermo Fisher Scientific, United States) was used as a negative control. Finally, the needle was kept in place for 5 min. Finally, we used medical bone wax to sealed the burr hole and the incision was closed with sutures.

### Real-Time Polymerase Chain Reaction (RT-PCR)

TRIzol reagent (Sigma–Aldrich, St. Louis, MO, United States) was applied to extract total RNA. Then, RNA (1 μg) from each sample was reverse-transcribed to cDNA by PrimeScript^TM^ RT reagent kit (Takara Bio Inc, Shiga, Japan). Afterward, qRT-PCR was conducted with SYBR^®^ Premix Ex Taq^TM^ (Takara Bio Inc, Shiga, Japan) on a 7300 Plus Read-Time PCR System (Thermo Fisher Scientific, Waltham, MA, United States). The cDNA was used as a template in a 20 ml reaction volume (10 μl of PCR mix, 5 pmol of forward and reverse primers, 1 μl cDNA template and proper volume of water), including a blank control (without template) in all experiments. The PCR reaction was performed as follows: initial denaturation at 94°C for 3 min; 45 cycles of denaturation at 94°C for 20 s, annealing at 60°C for 60 s, and elongation at 72°C for 45 s; and final elongation at 72°C for 3 min. Each sample was examined in triplicate. The primer sequences are as follows:

(1) ATF6: 5′-AAGTGAAGAACCATTACTTTATATC-3′ (forward) and 5′-TTTCTGCTGGCTATTTGT-3′ (reverse); (GenBank ID: 2138640)
(2) CHOP: 5′-GGAAGTGCATCTTCATACACCACC-3′ (for-ward) and 5′-TGACTGGAATCTGGAGAGAGCGAGGGC-3′ (reverse); (GenBank ID: 2138665)
(3) caspase-3: 5′-CGGACCTGTGGACCTGAAAA-3′ (forward) and 5′-TAGTAACCGGGTGCGTAGA-3′ (reverse); (GenBank ID: 2138666)
(4) β-actin: 5′-CACTGCCGCATCCTCTTCCT-3′ (forward) and 5′-AACCGCTCATTGCCGATAGTG-3′ (reverse); (GenBank ID: 2138668)

### Statistical Analysis

Results were displayed as mean ± SD. We performed the analysis with SPSS 22.0 software (IBM, United States). One-way ANOVA analysis of variance was applied for the comparison between different groups, with a *p* < 0.05 deeming to be statistically significant. However, we used non-parametric statistics to analyze data regarding the time-course of ATF6 as it did not pass the normality test.

## Results

### Physiological Data, BBB Integrity and Neurological Functions Assessment

During the surgery, we collected the physiological parameters, including body temperature, heart rate, blood pressure, blood glucose, PO2 and PCO2. No significant differences of physiological parameters were observed across each group (**Supplementary Tables III**, **IV**).

In order to assess the BBB permeability, brain water content and neurological functions at 24 h after ICH, two dosages of melatonin (100 mg/kg, 150 mg/kg) were administered intraperitoneally at 1 h after ICH. The induction of ICH significantly increased the brain water volume (*p* < 0.05 vs. sham). However, the administration of a high dose of melatonin was more significant in reducing brain water content at 24 h after ICH (*p* < 0.05 vs. ICH + vehicle, **Figure 2B**). The EB extravasation was increased in ipsilateral hemisphere of ICH compared with the rats in sham group (*p* < 0.05 vs. sham), while the rats receiving the higher dose of melatonin displayed a lower level of EB leakage (*p* < 0.05 vs. ICH + vehicle, **Figure 2C**). In addition, remarkable neurobehavioral function impairment was observed in ICH group while administration of the higher dose of melatonin significantly improved the neurological functions (*p* < 0.05, **Figure 2D**).

### Changes of the Expression of ATF6 and Its Role in Melatonin-Mediated Neuroprotection at 24 h After ICH

**FIGURE 3:**
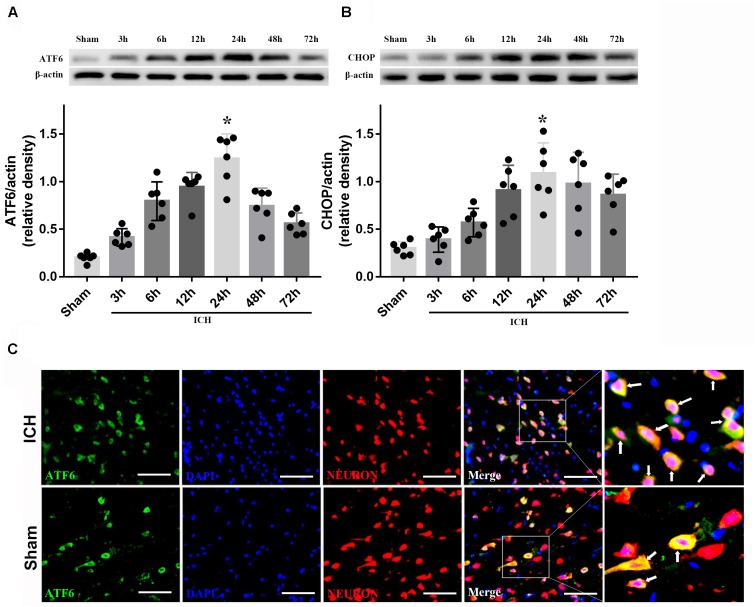
Expression of ATF6 and CHOP. **(A)** Time course of ATF6 in injured hemisphere after ICH; **(B)** time course of CHOP; *n* = 6 for each group. The bars represent the mean ± SD. ^∗^*p* < 0.05 vs. sham; **(C)** Representative microphotographs of immunofluorescence staining showing localization of ATF6 (green) and NeuN (red) in the perihematomal region after ICH (*N* = 2 for each group). Scale bar = 50 μm.

The results of time-course study showed that the level of ATF6 started to rise at 3 h and reached peak at 24 h after ICH (*p* < 0.05, **Figure 3A**). The expression of CHOP significantly increased at 12 h, and peaked at 24 h after ICH (*p* < 0.05, **Figure 3B**). Furthermore, the double immunostaining of ATF6 with NeuN in sham and ICH + vehicle groups showed that ATF6 was highly expressed in neurons (**Figure 3C**).

**FIGURE 4:**
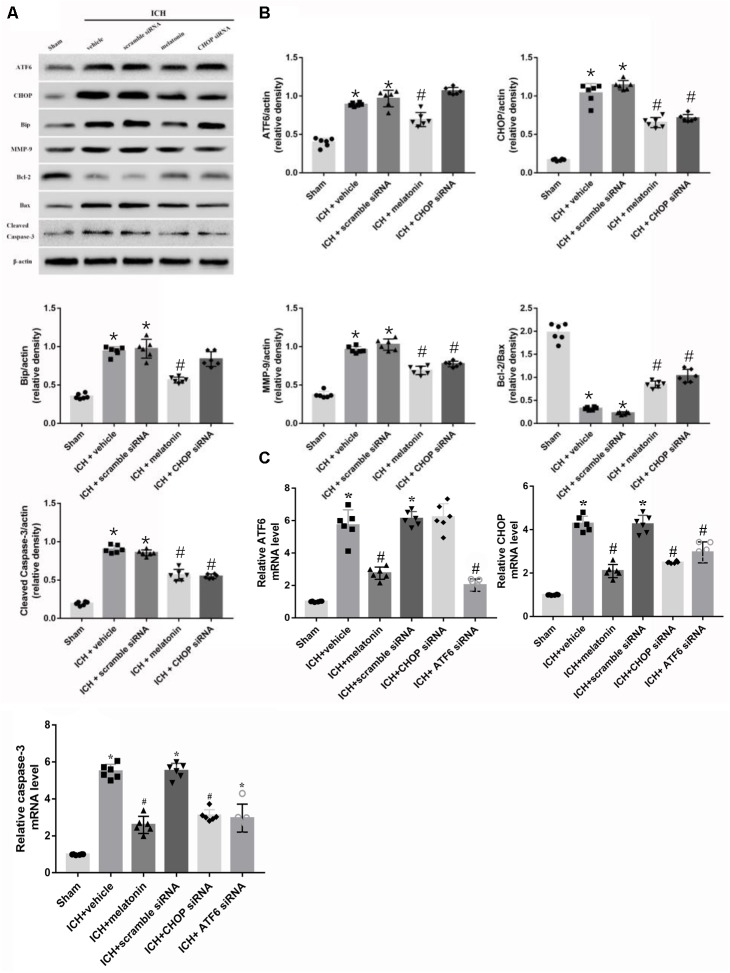
Intraperitoneal administration of melatonin Promotes Neuronal Survival at 24 h after ICH. **(A)** Representative Western blot images. **(B)** Quantitative analyses of ATF6, CHOP, Bip, MMP-9, Bcl-2, Bax, cleaved caspase-3; **(C)** Relative level of mRNAs of ATF6, CHOP and caspase-3. *n* = 6 for each group. The bars represent the mean ± SD. ^∗^*p* < 0.05 vs. sham, #*p* < 0.05 vs. ICH + vehicle.

**FIGURE 5:**
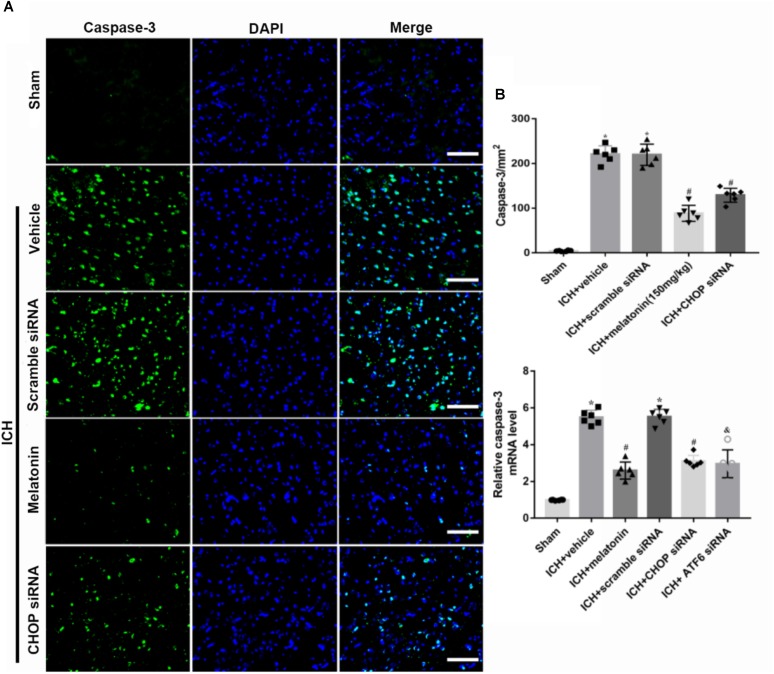
The administration of melatonin significantly decreased the number of caspase-3 and DAPI double-stained cells in the perihematomal region 24 h after ICH. **(A)** Representative microphotographs showed the co-localization of DAPI (blue) with caspase-3 (green)-positive cells in injured brain hemisphere at 24 h after ICH; **(B)** quantitative analysis of caspase-3 positive cells showed that melatonin decreased the number of apoptotic cells after ICH.

**FIGURE 6:**
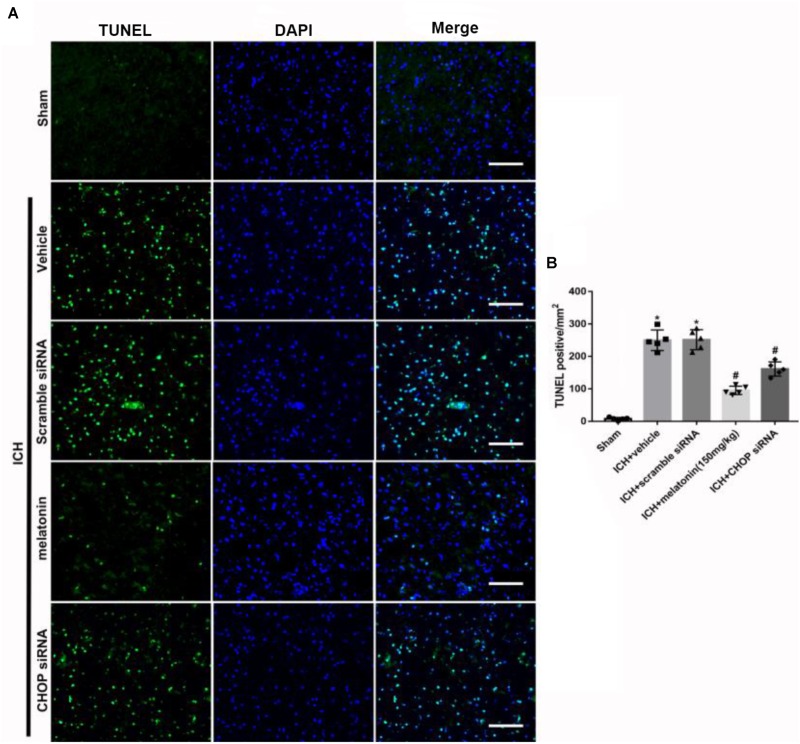
The administration of melatonin significantly decreased the number of TUNEL and DAPI double-stained cells in the perihematomal region 24 h after ICH. **(A)** Representative microphotographs showed the co-localization of DAPI (blue) with TUNEL (green)-positive cells in injured brain hemisphere at 24 h after ICH; **(B)** Quantitative analysis of TUNEL-positive cells showed that melatonin decreased the number of apoptotic cells after ICH. Scale bar = 100 μm. ^∗^*p* < 0.05 vs. sham, #*p* < 0.05 vs. ICH + vehicle.

Based on the neuroprotective effects of melatonin, which has been proved above, we chose high dose of melatonin for mechanism study. The results showed that administration of the high dose of melatonin significantly reduced the expression of ATF6 compared with ICH + vehicle group in both protein and mRNA levels (*p* < 0.05, **Figures 4A–C**). Additionally, the ratio of Bcl-2/Bax was significantly reduced, while the mRNA and protein level of cleaved caspase-3 was increased in ICH + vehicle group (*p* < 0.05, vs. sham). The administration of the higher dose of melatonin could significantly reverse these results (*p* < 0.05, vs. ICH + vehicle, **Figures 4A–C**). Furthermore, the administration of melatonin significantly reduced the expression level of MMP-9, which was increased after the introduction of ICH (*p* < 0.05, vs. ICH + vehicle, **Figures 4A,B**). The increase of MMP-9 further indicated the disruption of BBB except for the results from EB testing. The results of TUNEL staining suggested that the number of TUNEL positive cells and caspase-3 significantly increased at 24 h after ICH (*p* < 0.05, ICH vs. sham), whereas the number of TUNEL-positive and caspase-3 positive cells was obviously decreased after the administration of the higher dose of melatonin (*p* < 0.05, vs. ICH + vehicle, **Figures 5A,B**, **6A,B**).

### Role of Downstream CHOP in the ATF6-Mediated Pro-apoptotic Effects 24 h After ICH

**FIGURE 7:**
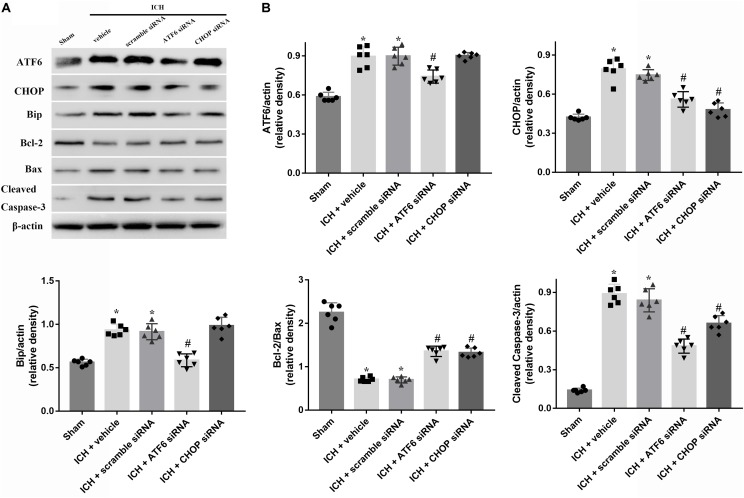
Application of ATF6 siRNA promotes neuronal survival at 24 h after ICH. **(A)** Representative Western blot images. **(B)** Quantitative analyses of ATF6, CHOP, Bip, Bcl-2, Bax, cleaved caspase-3. *n* = 6 for each group. The bars represent the mean ± SD. ^∗^*p* < 0.05 vs. sham, #*p* < 0.05 vs. ICH + vehicle.

In order to explore the role of CHOP in the ATF6 signaling pathway, ATF6 siRNA and CHOP siRNA were injected intracerebroventricularly at 48h before ICH. The results showed that the administration of ATF6 siRNA could significantly reduce the level of CHOP expression in both protein and mRNA levels, while CHOP siRNA had no effects on the expression of ATF6, which was elevated at 24 h after ICH (*p* < 0.05, vs. sham). However, the increased expression of CHOP induced by ATF6 at 24 h after ICH was significantly suppressed by CHOP siRNA (*p* < 0.05 vs. ICH + vehicle, **Figures 7A,B**). Meanwhile, the induction of ICH could increase the cell apoptosis by decreasing Bcl-2/Bax ratio and increasing cleaved caspase-3 expression (*p* < 0.05, sham vs. ICH + vehicle); However, these pro-apoptotic effects could be significantly reversed by CHOP siRNA (*p* < 0.05, vs. ICH + vehicle, **Figures 7A,B**).

## Discussion

In this study, we explored the neuroprotective effects of melatonin in a new mechanism mediated by suppression of ATF6/CHOP pathway in the experimental ICH of rats. The induction of ICH could significantly enhance the expression of ATF6, and its downstream effector, CHOP, both of which peaked at 24 h after ICH. ATF6 was expressed primarily in neurons. Up-regulation of ATF6 and CHOP expression increased neuronal apoptosis, which would be partially offset by administration of the higher dose of melatonin. Melatonin significantly improved the neurological functions, reduced brain edema, BBB disruption and neuronal apoptosis by suppressing the expression of ATF6 and CHOP in both protein and mRNA levels, increasing Bcl-2 expression, and reducing the levels of Bax and cleaved caspase-3. In addition, the inhibition of CHOP signaling with siRNA markedly decreased the pro-apoptotic effects of ATF6 induced by ICH.

Intracerebral hemorrhage is usually caused by ruptured vessels that are degenerated due to long-standing hypertension (Boulouis et al., 2017). The underlying mechanisms include the disturbance of ion hemostasis, the induction of oxidative stress, and apoptosis. The ER is an organelle which mainly manipulates protein synthesis and processing (Qiu et al., 2017). Insults that perturb ER function result in ER stress (Gong et al., 2017). It could be induced by inflammation, oxidative stress, mitochondrial calcium overloading (Louessard et al., 2017), and toxic glutamate release. Overactivation of ER stress leads to an accumulation of misfolded proteins in the ER. ATF6, a single-pass transmembrane protein, is the key response factor in the regulation of misfolded proteins. ER stress could significantly increase the activation of ATF6, which then translocates to the Golgi, where it is cleaved by the Golgi-localized proteases, SP1 and SP2 (Glembotski, 2014). The resulting 50 kD cytosolic fragment translocates to the nucleus, binds to elements in ATF6-responsive genes, resulting in the sustained reduction of global protein synthesis, thereby determining the fate of the cells (Stephens and Nicchitta, 2008; Xiong et al., 2017). In an *in vitro* study of hypoxic-ischemic encephalopathy (HIE), the cells suffering from hypoxia could have significantly increased levels of cleaved ATF6, which would promote apoptosis. The results demonstrated that ATF6 is an important factor in triggering HIE brain injury (Liu et al., 2015). In the present study, we determined the time course of the expression level of ATF6 and its downstream signal, CHOP, after ICH. The results were consistent with the abovementioned observations that protein levels of ATF6 and CHOP were significantly increased and peaked at 24 h after ICH. Based upon these results, the activity of ATF6 was strongly associated with increased pro-apoptotic markers in the experimental ICH model.

We further investigated the role of ATF6 in experimental ICH and its relationship with melatonin by intraperitoneal injection of it 1 h after ICH. The administration of melatonin could significantly reduce the protein and mRNA levels of ATF6 expression. In addition, the expression of Bcl-2 was up-regulated while Bax and cleaved caspase-3 levels were down-regulated. Colocalized immunofluorescence staining showed that neuronal apoptosis was significantly increased after ICH, while the administration of melatonin reversed the results. Furthermore, the induction of ICH significantly increased the level of EB and MMP-9, which was offset by the administration of melatonin. MMP-9, also known as type IV collagenase, belongs to the family of zinc-dependent metalloendo-peptidases. It could degrade the structures of cell junctions, like Claudin-5, ZO-1, et al., and destroy the BBB (Vafadari et al., 2016). The levels of EB and MMP-9 were increased, which indicated that the BBB was destroyed after ICH. However, the administration of melatonin restored the integrity of BBB. Additionally, melatonin could improve neurobehavioral deficits and reduce brain edema at 24 h after ICH. These results were consistent with what has been reported in the literature. The neuroprotective effects of melatonin have been verified in many studies. The mechanisms involved anti-inflammation, anti-oxidative stress, BBB protection and anti-apoptosis (Ramos et al., 2017; Wu et al., 2017). Recently, an increasing amount of studies have demonstrated roles of melatonin in the regulation of ER stress. Melatonin reduced cellular apoptosis and autophagy via suppression of ER stress in both *in vivo* and *in vitro* studies (Hosseinzadeh et al., 2016; Tungkum et al., 2017).

However, the role of ATF6 currently remains controversial. Contrary to our study, the activation of ATF6 was also reported to act as a cellular protective factor in many disease models. For example, in cultured cardiac myocytes, Peter J. and his colleagues demonstrated that adenovirus over-expressing ATF6 could protect cardiac myocytes from apoptosis by down-regulation of miR-455 and *calr* expression (Belmont et al., 2012). In addition, the activation of ATF6 branch of the UPR was also reported to improve the outcomes after cerebral ischemia(Yu et al., 2018). On the contrary, it has been demonstrated in a rat model of spinal cord injury, that knockout of ATF6 could protect against ER stress and promote oligodendrocyte precursor cell survival (Saraswat et al., 2018). Actually, the activation of ATF6 branch of UPR has dual roles in the regulation of cell survival. If the injury is mild and short, the activation of ATF6 could help to fold the proteins in a right way and decrease the ER stress, otherwise, the ATF6 would initiate the pro-apoptotic pathway. In response to the ER stress, ATF6 regulates the expression of several genes, the products of which can either relieve ER stress or trigger cell death if the stress cannot be relieved (DuRose et al., 2006). ICH is a subtype of stroke and characterized by high mortality and morbidity. The devastating outcome after ICH was mainly caused by direct damages to the brain parenchyma from blood accumulation and secondary injuries such as brain edema, BBB disruption, and inflammation, which leads to neuronal apoptosis or necrosis. The resulting accumulations of blood components, overproduced iron complexes, dysfunctional organelles, and other cytokines can disrupt normal protein folding, leading to activation of UPR/ER stress, which eventually leads to the irreversible disruption of brain parenchyma and massive cell death (Niu et al., 2017). The results of this study suggested that overactivated ER stress occurred at 24 h after ICH and suppression of ATF6 reduced neuronal apoptosis. In addition, Quan et al. (2015) found that melatonin could bind to its receptor and further suppress the activation of ER stress markers, including (Bip)/(GRP78), which is essential for the activation of ATF6. In the present study, the use of melatonin could significantly decrease the level of Bip and ATF6, suggesting that melatonin could suppress the activation of ATF6 via reducing the level of Bip (Quan et al., 2015).

We further explored the role of CHOP in melatonin-mediated neuroprotection. CHOP remains in low levels under normal conditions, but is greatly up-regulated in response to ER stress (Tabas and Ron, 2011). The active role of CHOP has been reported in many diseases. Miyazaki et al. (2011) demonstrated that CHOP deficiency could attenuate myocardial reperfusion injury by inhibiting cellular apoptosis and inflammation. In the rat model of ischemic stroke, suppression of CHOP expression could prevent ER stress overactivation and finally promote neuroprotection. Furthermore, Gotoh et al. (2002) explored the role of ATF6/CHOP pathway in the NO-mediated apoptosis in macrophages. They demonstrated that cell apoptosis induced by p50ATF6 could be inhibited by either a CHOP or ATF6 form (Gotoh et al., 2002). The mechanisms of CHOP-mediated apoptosis are reported to be the suppression of Bcl-2 expression and promotion of Bax expression both *in vivo* and *in vitro* (Wang et al., 1998; Sok et al., 1999; Fu et al., 2010). Based on the results aforementioned, CHOP acted as a pro-apoptotic factor mediated by ATF6 in the ER stress. In the present study, the results suggested that the level of CHOP significantly increased at 12 h and peaked at 24 h after ICH. However, the application of ATF6 siRNA or melatonin distinctly reduced both the protein and mRNA levels of CHOP expression, as well as the expression of Bax and cleaved caspase-3. Additionally, the pro-apoptotic characteristics of ATF6 induced by ICH could be partially reversed by the use of CHOP siRNA, which suggested that the CHOP also acts as a pro-apoptotic factor in the ATF6/CHOP pathway. All of the results suggested that melatonin exerts its neuroprotective role in ICH via suppression of ATF6 and CHOP expression.

Although this study verified the value of melatonin in a novel mechanism mediated via ATF6/CHOP signaling, some limitations could not be ignored. Firstly, melatonin could exert its neuroprotective effects in many pathways by the regulation of ER stress. This study only explored its role in the ATF6/CHOP pathway. Secondly, only the pro-apoptotic characteristic of ATF6 was evaluated in this study, without further investigation of its role in inflammation or autophagy. Therefore, studies focusing on further exploration of melatonin in ER stress and other characteristics of ATF6 in the experimental ICH model are needed.

## Ethics Statement

All experimental protocols were warranted by the ethics committee of Zhejiang University. The procedures were conducted according to NIH guidelines.

## Author Contributions

WX and TL designed the study. WX, JinZ, and XL completed the experiments. AS and LG performed the statistical analysis. WX and JinZ finished writing the manuscript. CL and XL revised the manuscript. JY and JiaZ participated in discussion development and provided expert guidance.

## Conflict of Interest Statement

The authors declare that the research was conducted in the absence of any commercial or financial relationships that could be construed as a potential conflict of interest.

## Abbreviations

ATF6: activating transcription factor 6
BBB: blood brain barrier
CHOP: CCAAT/enhancer-binding protein (C/EBP) homologous protein
EB: Evans blue
ER: endoplasmic reticulum
ICH: intracerebral hemorrhage
IRE1: inositol requiring kinase 1
NSS: Neurological Severity Score
PERK: protein kinase-like ER kinase
RT-PCR: reverse transcription-polymerase chain reaction
SD: Sprague–Dawley
TUNEL: Terminal deoxynucleotidyl transferase (TdT)-mediated dUTP nick end labeling
UPR: unfolded protein response
