# Supplementary material for: Melatonin Protects Against Neuronal Apoptosis via Suppression of the ATF6/CHOP Pathway in a Rat Model of Intracerebral Hemorrhage
Source: Front Neurosci. 2018 Sep 19;12:638. doi: 10.3389/fnins.2018.00638 (PMC6156428; doi:10.3389/fnins.2018.00638)
Supplement: Supplementary file 1 [file Table_1.DOCX]

**Supplemental TableⅠ.The animal usage.**

| Part | Groups | Brain Edema | EB | PCR | WB | IF | mortality(ICH) | excluded(ICH) | Sum |
| --- | --- | --- | --- | --- | --- | --- | --- | --- | --- |
| Exp. 1 | Sham | 6 | 6 | - | - | - | 35/242(14.46%) | 5 | 291 |
|  | ICH | 6 | 6 | - | - | - |  |  |  |
|  | ICH + vehicle | 6 | 6 | - | - | - |  |  |  |
|  | ICH + melatonin (100mg/kg) | 6 | 6 | - | - | - |  |  |  |
|  | ICH + melatonin (150mg/kg) | 6 | 6 | - | - | - |  |  |  |
| Exp. 2 | Sham | - | - | - | 6 | 2 |  |  |  |
|  | ICH (3h, 6h, 12h, 24h, 48h, 72 h) | - | - | - | 36 | 2 |  |  |  |
| Exp. 3 | Sham | - | - | 6 | 6 | 5 |  |  |  |
|  | ICH + vehicle | - | - | 6 | 6 | 5 |  |  |  |
|  | ICH + scramble siRNA | - | - | 6 | 6 | 5 |  |  |  |
|  | ICH + melatonin | - | - | 6 | 6 | 5 |  |  |  |
|  | ICH+ CHOP siRNA | - | - | 6 | 6 | 5 |  |  |  |
| Exp. 4 | Sham | - | - | 6 | 6 | - |  |  |  |
|  | ICH + vehicle | - | - | 6 | 6 | - |  |  |  |
|  | ICH + scramble siRNA | - | - | 6 | 6 | - |  |  |  |
|  | ICH+ ATF6 siRNA | - | - | 6 | 6 | - |  |  |  |
|  | ICH+ CHOP siRNA | - | - | 6 | 6 | - |  |  |  |
|  | Subtotal | 30 | 30 | 60 | 102 | 29 | 35 | 5 |  |

ICH, intracerebral hemorrhage; PCR, polymerase chain reaction; EB, Evans Blue; WB, western blot; IF, immunofluorescence.

| **Supplemental Table Ⅱ. Neurological Severity Scores (NSS)** | | |
| --- | --- | --- |
|  | **Items** | **Score** |
| **raising rat by tail (normal=0; maximum=3)** | | (3) |
|  | flexion of forelimb | 1 |
|  | flexion of hindlimb | 1 |
|  | head moved >10° to vertical axis within 30s | 1 |
|  | placing rat on floor (normal; maximum=3) | (3) |
|  | normal walk | 0 |
|  | inability to walk straight | 1 |
|  | circling toward paretic side | 2 |
|  | falls down to paretic side | 3 |
| **sensory tests (normal=0; maximum=2)** | | (2) |
|  | placing test (visual and tactile test) | 1 |
|  | proprioceptive test (deep sensation) | 1 |
| **beam balance tests (normal=0; maximum=6)** | | (6) |
|  | balances with steady posture | 0 |
|  | grasps side of beam | 1 |
|  | hugs beam and 1 limb falls down from beam | 2 |
|  | hugs beam and 2 limbs falls down from beam, or spins on beam(>60s) | 3 |
|  | attempts to balance on beam but falls off(>40s) | 4 |
|  | attempts to balance on beam but falls off(>60s) | 5 |
|  | falls off; no attempt to balance or hang on to beam(<20s) | 6 |
| **reflex absence and abnormal movements** | | (4) |
|  | pinna reflex (head shake when auditory meatus is touched) | 1 |
|  | corneal reflex (eye blink when cornea is lightly touched with cotton) | 1 |
|  | startle reflex (motor response to a brief noise) | 1 |
|  | seizure, myoclonus, myodystony | 1 |
| **maximum points** | | (18) |

| **Supplemental Table Ⅲ. Rats physical indices before surgeries.** | | | | | | | | |
| --- | --- | --- | --- | --- | --- | --- | --- | --- |
| **Group** | **Rats used** | **T(℃)** | **HR(/min)** | **BP(mmHg)** | **BG(mmol/L)** | **PO2(mmHg)** | **PCO2(mmHg)** |  |
| **Sham** | **51** | 37.4±0.22 | 328±6.2 | 133±0.53 | 6.6±0.25 | 76±5.1 | 37±2.0 |  |
| **ICH** | **50** | 37.6±0.15 | 335±7.5 | 137±1.2 | 6.7±0.49 | 74±4.6 | 39±1.9 |  |
| **ICH + vehicle** | **43** | 37.3±0.21 | 356±6.9 | 142±1.1 | 6.6±0.33 | 75±4.9 | 41±2.4 |  |
| **ICH + melatonin** | **43** | 37.4±0.17 | 333±7 | 135±0.88 | 6.5±0.27 | 72±3.8 | 35±1.6 |  |
| **ICH + scramble siRNA** | **29** | 37.5±0.28 | 367±7.8 | 125±0.92 | 6.6±0.33 | 73±5.9 | 44±2.4 |  |
| **ICH + ATF6 siRNA** | **12** | 37.5±0.17 | 329±4.9 | 135±0.58 | 6.6±0.30 | 74±6.2 | 40±2.6 |  |
| **ICH + CHOP siRNA** | **29** | 37.6±0.16 | 338±6.8 | 126±0.7 | 6.4±0.25 | 78±5.6 | 39±2.5 |  |

ICH, intracerebral hemorrhage; T, temperature; HR, heart rate; BP, blood pressure; BG, blood glucose.

| **Supplemental Table Ⅳ.** **Rats physical indices after surgeries.** | | | | | | | |
| --- | --- | --- | --- | --- | --- | --- | --- |
| **Group** | **Rats used** | **T(℃)** | **HR(/min)** | **BP(mmHg)** | **BG(mmol/L)** | **PO2(mmHg)** | **PCO2(mmHg)** |
| **Sham** | **51** | 37.6±0.23 | 358±8.5 | 134±0.66 | 6.5±0.31 | 72±4.8 | 35±2.1 |
| **ICH(24h)** | **50** | 37.9±0.19 | 347±7.5 | 131±1.1 | 6.4±0.37 | 70±4.5 | 42±2.5 |
| **ICH + vehicle** | **43** | 37.8±0.21 | 363±8.1 | 128±0.91 | 6.6±0.41 | 74±5.1 | 39±2.2 |
| **ICH + melatonin** | **43** | 37.6±0.18 | 359±7.4 | 132±0.85 | 6.7±0.39 | 72±3.3 | 45±2.6 |
| **ICH + scramble siRNA** | **29** | 37.8±0.26 | 355±8.9 | 126±0.76 | 6.5±0.27 | 76±5.5 | 41±1.9 |
| **ICH + ATF6 siRNA** | **12** | 37.9±0.15 | 366±9.1 | 133±0.63 | 6.6±0.35 | 71±4.4 | 42±2.7 |
| **ICH+CHOP siRNA** | **29** | 37.7±0.20 | 341±7.2 | 128±0.58 | 6.5±0.45 | 72±5.5 | 38±2.2 |

ICH, intracerebral hemorrhage; T, temperature; HR, heart rate; BP, blood pressure; BG, blood glucose.
